# Showing Up is Still Resistance: Contextualizing Black Trainee Resistance in Medical Education

**DOI:** 10.5334/pme.1972

**Published:** 2025-07-23

**Authors:** Oriaku A. Kas-Osoka, Patricia Poitevien

**Affiliations:** 1 Department of Pediatrics at the University of Arkansas for Medical Sciences and Arkansas Children’s Hospital in Little Rock, Arkansas, USA; 2 Department of Pediatrics at Brown University and Hasbro Children’s Hospital in Providence, Rhode Island, USA

## Abstract

This commentary amplifies the contributions of Horton et al.’s study on Black physician trainees’ professional resistance, situating it within the broader landscape of health professions education (HPE). As Black women physician-educators, we reflect on the profound resonance of the study’s themes—resistance as survival, as existence, and as professionalism. Horton et al. employ Endarkened Storywork and Black quilting to not only document Black trainee experiences but to disrupt conventional research paradigms that marginalize their truths. This commentary argues that their work challenges the field to reimagine equity by centering marginalized voices, redefining resistance as a daily, embodied practice, and questioning research frameworks rooted in whiteness. We emphasize the urgency of methodological transformation in HPE and the ethical imperative to design and interpret data in ways that affirm rather than exploit minoritized communities. By framing the acts of showing up, mentoring, and refusing assimilation as legitimate resistance, this work redefines professionalism and provides a roadmap for institutional accountability. To advance equity, educators and researchers must not only study resistance—they must practice it.

## Commentary

This commentary amplifies and contextualizes Horton and colleagues’ vital contribution to health professions education (HPE). As two Black women physicians and educators navigating institutions shaped by structural racism and white normativity, we read this study with both deep resonance and firm resolve. We know what it means to perform under surveillance, to resist silently in spaces not built for us, and to build community while battling erasure.

The field of HPE stands at a crossroads. As sociopolitical backlash threatens the progress made in diversity, equity, and inclusion (DEI), the imperative to reimagine—not just preserve—equity in medical training has never been more urgent [1,2,3]. In this context, Horton et al.’s study, *“I Have to Resist Simply to Exist: Black Physician Trainees’ Experiences of Professional Resistance,”* is both timely and transformative. Through their integration of Endarkened storywork, Re-storying, and Black quilting [4,5], they do more than document Black trainees’ experiences—they expand the epistemological possibilities of HPE research.

This work carries urgent implications for the field. It calls on scholars to interrogate our methodologies and examine how dominant research paradigms, often rooted in colonial logics, perpetuate harm. Are we centering the voices and experiences of minoritized communities, or merely studying them from a distance? Horton et al. provide a clear model for researching *with*, rather than *on*, marginalized communities.

Many research frameworks—even critical race theory—are still grounded in white academic traditions. They often demand that Black experiences be legible only through the lens of whiteness. HPE research is no exception [6]. As scholars whose work explores how underrepresented in medicine (UIM) pediatric residents navigate wellness and professional identity, we view Horton et al.’s intervention as refreshing and necessary [7,8]. It affirms what many of us have long sensed: even our most “progressive” research tools can reinscribe the power structures we seek to dismantle.

What makes this study groundbreaking is not only its content, but the way that content is revealed. Endarkened storywork offers a methodology of nurture, not just critique. Rather than force narratives into thematic codes, this approach allows stories to emerge as “quilting blocks” [5]: imperfect, layered, and deeply human. Scraps, seams, and threads are not anomalies—they are meaning.

Our research along with the research of others highlights how UIM trainees bear the weight of “double and triple duty”: healer, educator, and racial representative [1,3,9,10,11]. Horton et al.’s participants echo this burden, describing an existence threaded with emotional exhaustion, collective responsibility, and the relentless drive to create space for those who will follow. This is not incidental. It is survival. It is resistance.

For Black trainees, *existence is resistance*—a theme that pulses through every line of this study. This resistance is not metaphorical. It is material, emotional, and institutional. Horton et al. show how resistance is not episodic or elective through rich participant narratives and a textured analytic lens. It is ever-present, woven into the daily acts of showing up with textured hair, speaking in a voice that might be policed, advocating for patients in biased systems, and daring to dream beyond survival. These are not isolated incidents—they are collective acts of refusal. To recognize existence as resistance permits a radical reframing of the experience of UIM trainees in the clinical learning environment. As educators, advisors, and mentors, our failure to grasp this context, by virtue of how this data has been historically collected and disseminated, represents an abdication of our responsibilities to the learner.

This reframing demands a shift in how we approach structural change in graduate medical education. Reform is not an optional initiative—it is an ethical obligation. These findings must inform everything from mentorship programs to curriculum development and institutional policy [7]. It is not enough to ask how many Black trainees are in our programs; we must ask *how* they are being asked to show up, perform, and persist.

One of the study’s most powerful contributions is its articulation of *racial battle fatigue*—not as an abstract term, but as a lived reality. The participants describe constantly being judged as proxies for an entire race, with any misstep endangering future access for others [8,12]. This burden erodes wellness, undermines confidence, and drives many talented Black trainees away from academic medicine altogether [8]. It is worsened when institutions conflate diversity with equity or assume that recruitment alone suffices [9].

This study echoes the work of many who have called for values-aligned, anti-racist medical education [7,9]. When trainees resist by mentoring others, advocating for culturally responsive care, and imagining themselves as future faculty or deans, they are engaging in legacy work. That, we believe, is the highest form of professionalism and should be recognized as such.

To show up, to speak up, to stay—is resistance. But so too is saying no. No to metrics that erase. No to professionalism standards steeped in whiteness. No to DEI efforts that ask for our stories but not our leadership. As Horton et al. remind us, it is not enough to publish papers about equity—we must model equity in how we design our studies, choose our collaborators, and share our platforms.

If we are serious about equity in health professions education, we must confront our own blind spots and commit to using tools like those offered by Horton et al., not as optional add-ons, but as essential instruments in transforming how we see, support, and shape our learners.
